# Implementing a Candida auris Screening Program at a Comprehensive Cancer Center

**DOI:** 10.1017/ash.2025.425

**Published:** 2025-09-24

**Authors:** Adina Feldman, Micah Bhatti, Rebecca Poths, Jane Powell, Guy Handley, Amy Spallone

**Affiliations:** 1 MD Anderson Cancer Center; 2 M.D. Anderson Cancer Center; 3 Univ of Texas MD Anderson Cancer Center; 4 MD Anderson Cancer Center; 5 MD Anderson Cancer Center; 6 MD Anderson Cancer Center

## Abstract

**Background:** Candida auris (C. auris) is a resistant fungal pathogen that persists in the hospital environment and poses a significant infection risk, particularly to immunocompromised patients. Early detection and infection control are vital for patient safety as C. auris may spread between patients in healthcare settings through contact with contaminated surfaces. The Centers for Disease Control and Prevention (CDC) and Texas Department of State Health Services (Texas DSHS) recommend screening high-risk patients, including those previously hospitalized abroad, in rehabilitation or long-term care facilities, or those with indwelling medical devices, mechanical ventilation, immunocompromised conditions, or colonization by other multidrug-resistant organisms. We developed a C. auris screening program based on these guidelines, local epidemiology, patient risk factors, and facility characteristics. **Methods:** An initial point prevalence survey was conducted to identify our high-risk patient population, but no positive screening results were found during our pilot study. However, a retrospective review of patients with clinical C. auris infection revealed 53% (8/15) had transferred into our center from other healthcare facilities. A targeted screening program of transfer patients was developed, and a needs assessment identified gaps in infection prevention practices, staff knowledge, and laboratory capabilities. Patients meeting screening criteria had composite swabs collected from axilla and groin creases, which were sent to an external lab for C. auris PCR testing. A tracking system was established for patients, results, and newly identified infections. Newly identified colonization cases received targeted infection control measures and were placed on isolation. **Results:** During the first four months of the screening protocol, 588 transfer patients have been screened, reflecting 5.47% of all admissions. We identified ten positive colonization cases, yielding a 1.82% positivity rate. In addition, two C. auris infections were identified. **Conclusion:** Overall, our C. auris colonization rates are low. This may reflect differences in patient population or screening practices. Identifying ten colonized patients through our screening protocol enabled timely isolation and targeted infection control measures, preventing possible horizontal transmission in a high-risk patient population. Ongoing monitoring and evaluation will inform our screening practices and enhance our ability to respond to outbreaks. As the prevalence of C. auris continues to grow, our screening program will continue to provide a proactive approach to managing this growing threat to our highly vulnerable patient population.

